# Mitochondrial glutamine metabolism via GOT2 supports pancreatic cancer growth through senescence inhibition

**DOI:** 10.1038/s41419-017-0089-1

**Published:** 2018-01-19

**Authors:** Seungyeon Yang, Sunsook Hwang, Minjoong Kim, Sung Bin Seo, Jeong-Hwa Lee, Seung Min Jeong

**Affiliations:** 1 Department of Biochemistry, Institute for Aging and Metabolic Diseases, College of Medicine, The Catholic University of Korea, 222, Banpo-daero, Seocho-gu, Seoul, 06591 Republic of Korea; 2 Institute for Aging and Metabolic Diseases, College of Medicine, The Catholic University of Korea, 222, Banpo-daero, Seocho-gu, Seoul, 06591 Republic of Korea

## Abstract

Cellular senescence, which leads to a cell cycle arrest of damaged or dysfunctional cells, is an important mechanism to restrain the malignant progression of cancer cells. Because metabolic changes underlie many cell-fate decisions, it has been suggested that cell metabolism might play key roles in senescence pathways. Here, we show that mitochondrial glutamine metabolism regulates senescence in human pancreatic ductal adenocarcinoma (PDAC) cells. Glutamine deprivation or inhibition of mitochondrial aspartate transaminase (GOT2) results in a profound induction of senescence and a suppression of PDAC growth. Glutamine carbon flow through GOT2 is required to create NADPH and to maintain the cellular redox state. We found that elevated reactive oxygen species levels by GOT2 knockdown lead to the cyclin-dependent kinase inhibitor p27-mediated senescence. Importantly, PDAC cells exhibit distinct dependence on this pathway, whereas knockdown of GOT2 did not induce senescence in non-transformed cells. The essentiality of GOT2 in senescence regulation of PDAC, which is dispensable in their normal counterparts, may have profound implications for the development of strategies to treat these refractory cancers.

## Introduction

Pancreatic ductal adenocarcinoma (PDAC), the most common type of pancreatic cancer, is one of the most malignant cancers^1^. PDAC exhibits acute resistance to all form of therapy, including conventional chemotherapy, radiotherapy and targeted agents, which leads to the dismal prognosis of PDAC patients^2^. The profound resistance to treatments indicates that these refractory cancers may have altered cell survival pathways and aberrant metabolism. Thus, it is critical to identify new therapeutic targets for PDAC.

Cellular senescence is a state of growth arrest in response to various cellular stress stimuli including replicative cell culture, oxidative stress, DNA damage and oncogene activation^3,4^. Senescence has been proposed to be an important tumor-suppressive mechanism. The premature senescence induction by tumor suppressors was observed in multiple cancer models^5,6^. Mutations or dysregulation of senescence regulators, including p53, p21, p16, and retinoblastoma protein (Rb), are frequently observed in many human cancers and strongly correlate with a worse prognosis^6,7^. Because accruing evidence suggests that metabolic regulation has a predominant role for determining cellular states, including proliferation and cell cycle arrest, it is not surprising that cell metabolism contributes to cellular senescence.

The dysregulation of cell metabolism is a defining feature of cancer cells. In PDAC, oncogenic KRAS, serving a critical role in PDAC initiation and maintenance, mediates this reprogramming of cellular energy metabolism to support its growth and survival^8^. Interestingly, PDAC cells exhibit a distinct glutamine (Gln) metabolism. Whereas many cancer cells rely on deamination of Gln-derived glutamate to replenish mitochondrial carbon pool, PDAC use Gln-derived aspartate (Asp) to maintain the cellular redox state, which is essential for PDAC growth^9^. Moreover, our previous work demonstrated that enhanced mitochondrial glutamine anaplerosis suppresses PDAC growth^10^. However, the importance of Gln metabolism in PDAC senescence is not well elucidated.

Due to the pivotal role of Gln metabolism as an important regulator of cellular redox balance and multiple cell fate determination, we sought to specifically probe the role of mitochondrial Gln pathways in regulating pancreatic cancer growth and senescence.

## Results

### Mitochondrial glutamine metabolism suppresses cellular senescence in PDAC

Increasing evidence demonstrates that many cancer cells use Gln to support their energetic and synthetic needs^11,12^. However, the role of Gln metabolism in cellular senescence is not well determined. Recently, we and others reported that Gln is critical for PDAC growth and survival^9,10^. Thus, we sought to explore the functional role of Gln metabolism in PDAC senescence.

**Fig. 1:**
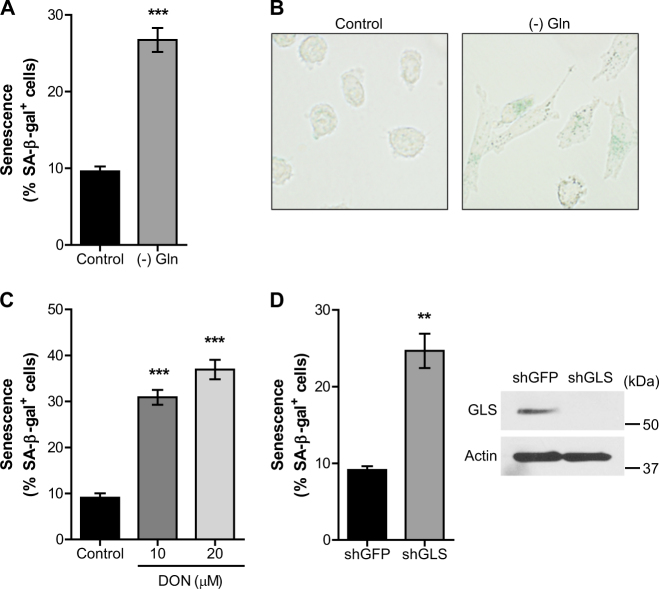
The Inhibition of mitochondrial glutamine metabolism induces senescence in PDAC cells **a**, 8988T cells were plated in complete media which was replaced the following day with Gln-free medium. Percentages of SA-β-gal positive cells are shown. **b**, Representative images of SA β-gal staining for 8988T cells in the presence or absence of Gln. **c**, Percentages of SA-β-gal positive cells in 8988T cells treated with increasing concentrations of DON. **d**, Percentages of SA-β-gal positive cells in control (shGFP) and GLS knockdown (shGLS) 8988T cells (left). Western blot confirmed knockdown of GLS expression (right). β-actin serves as a loading control. All error bars ± SEM. **p < 0.01 and ***p < 0.001

We first noticed that Gln deprivation caused a profound increase in 8988T PDAC cells expressing senescence-associated β-galactosidase (SA-β-Gal) (Fig. 1a). The induction of senescence in these cells was also indicated by large and flattened cellular morphology (Fig. 1b). Because proliferating cells use precursors derived from tricarboxylic acid (TCA) cycle, replenishment of the mitochondrial carbon pool is required for the maintenance of mitochondrial integrity and function^12^. Gln anaplerosis is essential to provide a carbon source to the TCA cycle. To assess the role of mitochondrial Gln metabolism in senescence, we treated cells with 6-diazo-5-oxo-L-norleucin (DON), an inhibitor of glutaminase (GLS). GLS is the first required enzyme for mitochondrial Gln anaplerosis. Notably, GLS inhibition significantly induced senescence in 8988T PDAC cells (Fig. 1c and S1A). Consistent with these results, GLS knockdown by using short hairpin RNAs (shRNAs) also strongly elicited senescence (Fig. 1d and S1B). Thus, these results demonstrate that mitochondrial Gln metabolism is essential for senescence regulation in PDAC cells.

### Glutamine metabolism through GOT2 regulates senescence in PDAC

**Fig. 2:**
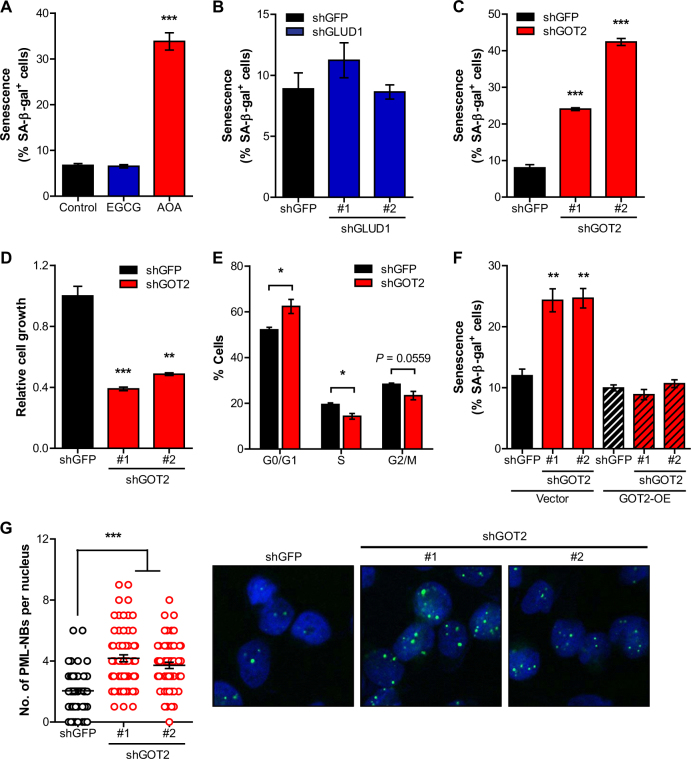
Mitochondrial transaminase GOT2 is responsible for the regulation of PDAC senescence **a**, Senescence induction of 8988T cells treated with EGCG (50 μM) or AOA (0.5 mM). **b** and **c**, Senescence induction of 8988T cells expressing a control shRNA (shGFP) or two independent shRNAs targeting GLUD1 **b**, or GOT2 **c**. **d**, Relative proliferation of 8988T cells expressing a control shRNA or shRNAs to GOT2. **e**, Percentage of cells in G0/G1, S and G2/M phases in 8988T cells expressing a control shRNA or a shRNA to GOT2. **f**, Percentages of SA-β-gal positive cells in GOT2 overexpressed (GOT2-OE) 8988T cells expressing a control shRNA or shRNAs to GOT2. **g**, Numbers of promyelocytic leukemia nuclear bodies (PML-NBs; left) and immunofluorescent staining using nuclear (DAPI; blue) and PML (green) in 8988T cells expressing a control shRNA or shRNAs to GOT2 (right).All error bars ± SEM. *p < 0.05, **p < 0.01 and ***p < 0.001

In mitochondria, Gln is metabolized via GLS to glutamate and ammonia (NH_4_^+^) and further catabolized to the TCA cycle intermediate α-ketoglutarate (αKG) via glutamate dehydrogenase (GLUD1) or transaminases to fuel mitochondrial carbon pool. To examine the mechanisms involved in the regulation of senescence in PDAC, we treated cells with either epigallocatechin gallate (EGCG), a GLUD1 inhibitor, or aminooxyacetate (AOA), a transaminase inhibitor. Whereas EGCG treatment had no effect on senescence, AOA treatment markedly induced senescence (Fig. 2a and S2A). To further confirm that mitochondrial transaminases regulate PDAC senescence, we reduced the levels of GLUD1 or mitochondrial aspartate transaminase (GOT2), highly upregulated in PDAC compared to other cancers^13^, by using shRNA constructs. Concordant with previous results, the levels of the SA-β-Gal positive cells upon GLUD1 knockdown were comparable with control (shGFP) (Fig. 2b and S2B). Conversely, GOT2 knockdown significantly elicited senescence in PDAC cells (Fig. 2c) and also resulted in morphological changes (Fig. S2C), impaired PDAC growth (Fig. 2d) and accumulation of cells in the G1 phase (Fig. 2e). Next, we found that overexpression of human GOT2 in PDAC cells can rescue the induction of senescence by GOT2 knockdown (Fig. 2f and S2D). Moreover, the induction of senescence upon GOT2 knockdown was also indicated by the accumulation of promyelocytic leukemia protein nuclear bodies (Fig. 2g). However, we observed no obvious increase in cell death under these conditions (Fig. S2E). Finally, we examined the involvement of another mitochondrial glutamate-dependent transaminase, pyruvate transaminase (GPT2), in PDAC senescence. We found that GPT2 knockdown did not affect senescence in PDAC cells (Fig. S2F and G). Together, these data demonstrate that mitochondrial transaminase GOT2 regulates senescence in PDAC cells.

### GOT2 is essential for sustaining growth and suppressing senescence in PDAC

**Fig. 3:**
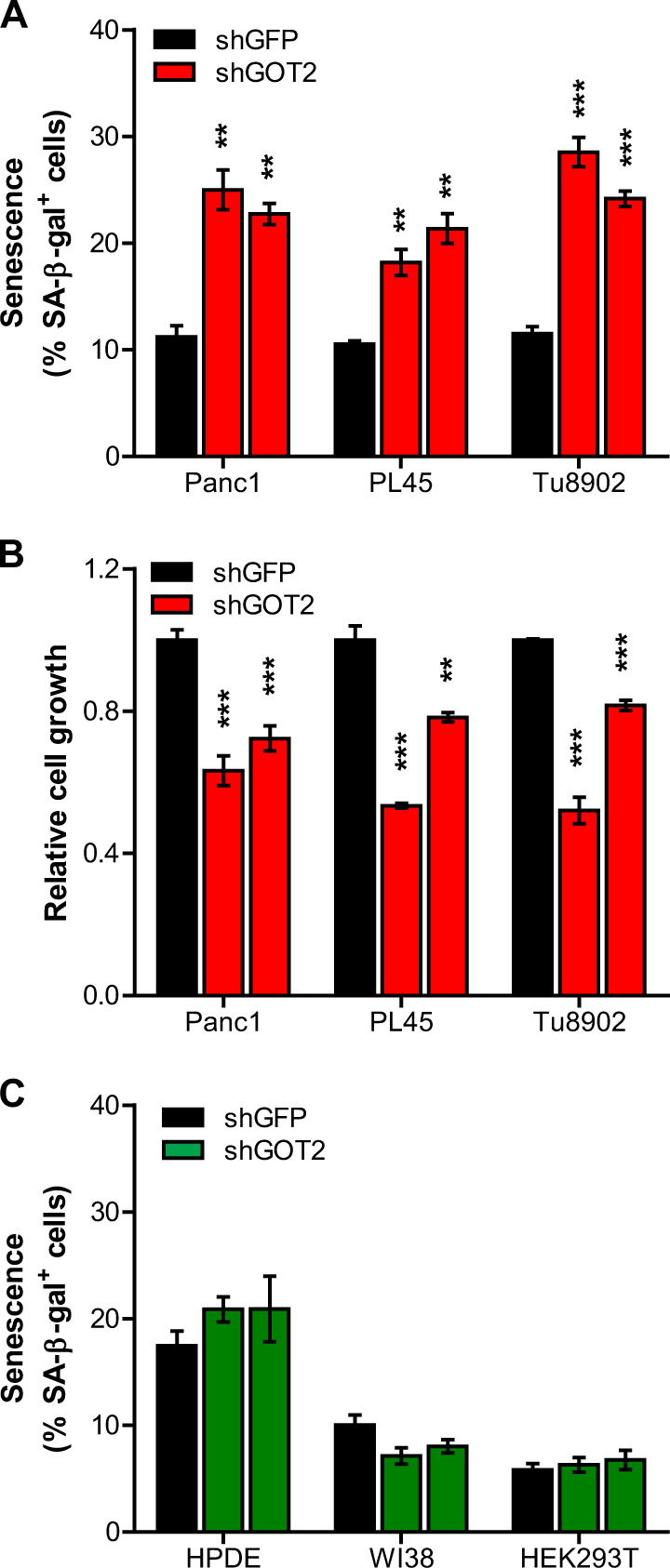
GOT2 regulates senescence in PDAC cells but not in normal cells **a** and **b**, Senescence induction **a**, and relative proliferation **b**, of PDAC cell lines (Panc1, PL45 and Tu8902) expressing a control shRNA (shGFP) or two independent shRNAs to GOT2. **c**, Percentages of SA-β-gal positive cells in HPDE, WI38 and HEK293T cells expressing a control shRNA (shGFP) or two independent shRNAs to GOT2. All error bars ± SEM. **p < 0.01 and ***p < 0.001

To further confirm the essential role of GOT2, we assessed whether GOT2 regulates senescence in multiple PDAC cells. Indeed, GOT2 knockdown significantly elicits senescence in all three tested PDAC lines (Fig. 3a and S3A). In line with these results, GOT2 inhibition also impaired the growth of these cells (Fig. 3b). Recent evidence suggests that PDAC cells have a greater reliance on Gln-derived Asp, whereas normal cells are dependent on GLUD1-mediated Gln pathway to fuel the TCA cycle^9^. Thus, we tested whether GOT2 regulates senescence in normal cells. Remarkably, in contrast to PDAC, GOT2 knockdown had only modest effects on senescence in non-transformed human pancreatic ductal cells (HPDEs) (Fig. 3c and S3B). Moreover, we observed similar results with human fibroblasts (WI-38) and human embryonic kidney 293 (HEK293T) cells. Thus, these results demonstrate that the Gln pathway mediated by GOT2 plays an important role in regulation of senescence in PDAC but not in non-transformed cells.

### GOT2 represses senescence by maintaining cellular redox balance

**Fig. 4:**
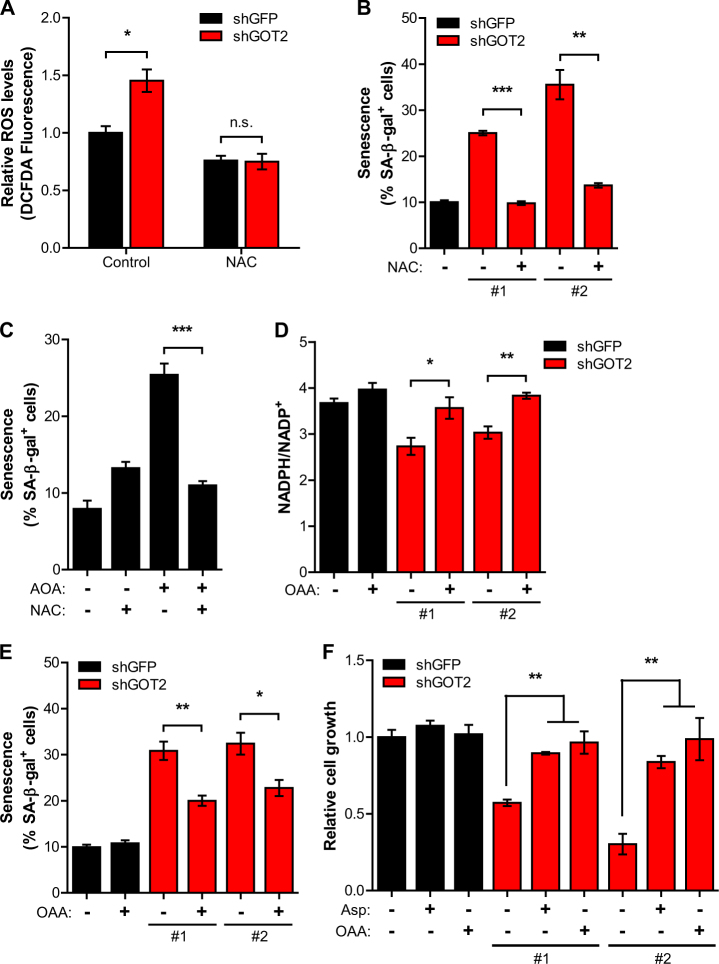
GOT2 suppresses PDAC senescence by regulating cellular ROS **a**, Relative ROS levels in 8988T cells expressing a control shRNA (shGFP) or a shRNA to GOT2 with or without NAC (10 mM). **b**, Senescence induction of 8988T cells expressing a control shRNA or two independent shRNAs to GOT2 supplemented with or without NAC. **c**, Senescence induction in control or AOA treated 8988T cells cultured with or without NAC. **d**, NADPH/NADP^+^ ratio in 8988T cells expressing a control shRNA or shRNAs to GOT2 supplemented with or without OAA (4 mM). **e** and **f**, Senescence induction **e**, and relative proliferation **f**, of 8988T cells expressing a control shRNA or two independent shRNAs to GOT2 supplemented with or without OAA (4 mM, or Asp (4 mM. All error bars ± SEM. *p < 0.05, **p < 0.01 and ***p < 0.001

In PDAC cells, the majority of aspartate (Asp) is converted from Gln and GOT2 knockdown markedly decreases Gln-derived Asp^9^. Indeed, we found that knockdown of GOT2 results in significant decreases in Asp and oxaloacetate (OAA) in PDAC cells (Fig. S4A). Because this Gln-derived Asp plays an essential role in the maintenance of cellular redox homeostasis in PDAC cells^9^, we next investigated whether GOT2 directly modulates cellular reactive oxygen species (ROS) production. Indeed, GOT2 knockdown cells exhibited significantly increased ROS levels, whereas the antioxidant *N*-acetylcysteine (NAC) treatment completely abrogated the increased ROS production (Fig. 4a). Given that increased ROS levels can contribute to induction of senescence^4,14^, we reasoned that the mechanism by which GOT2 regulates PDAC senescence involves ROS. To test this idea, we treated cells with NAC in order to probe the model that suppressing ROS could block the senescence induction by GOT2 knockdown. Notably, we observed that NAC treatment reduced senescence to comparable levels in control and GOT2 knockdown cells (Fig. 4b and S4B). Likewise, we also obtained similar results in AOA treatment-induced senescence (Fig. 4c and S4C).

Gln-derived Asp is metabolized by cytosolic GOT1 into OAA and then converted into malate, which is oxidized by malic enzyme (ME1) to produce NADPH, providing the reducing power to maintain redox homeostasis^9,15^. We found that GOT2 knockdown decreased the cellular NADPH/NADP^+^ ratio and the addition of OAA was able to rescue this ratio (Fig. 4d), suggesting that the GOT2-mediated conversion of Gln is important for the maintenance of ROS levels in PDAC cells. As further confirmation of the importance of this GOT2-mediated Gln pathways in PDAC senescence, we assessed the levels of SA-β-Gal positive cells in the presence of downstream metabolites of GOT2, such as Asp or OAA. Importantly, senescence induced by GOT2 knockdown was significantly reduced upon supplementation with OAA or Asp (Fig. 4e, S4D,E and F). Moreover, the addition of OAA or Asp rescued the growth suppression of GOT2 knockdown cells (Fig. 4f). Taken together, these results illustrate that GOT2 has a critical role in maintaining redox homeostasis, which is associated with modulation of cellular senescence in PDAC cells.

### p27 mediates GOT2 knockdown-induced senescence

**Fig. 5:**
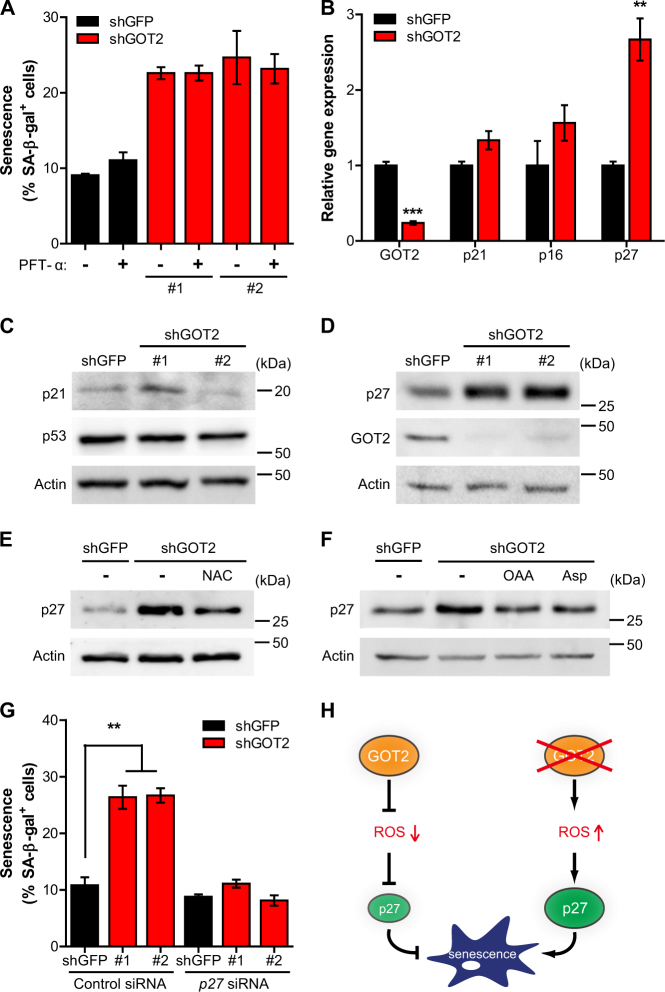
p27 is responsible for GOT2 knockdown-mediated senescence induction in PDAC cells **a**, Percentages of SA-β-gal positives cells in 8988T cells expressing a control shRNA or two independent shRNAs to GOT2 supplemented with or without PFT-α. **b**, Relative mRNA levels of indicated genes in 8988T cells expressing a control shRNA or a shRNA to GOT2. **c**, p21 and p53 protein levels in whole-cell lysates from 8988T cells expressing a control shRNA or shRNAs to GOT2. β-actin serves as a loading control. **d**, The effect of GOT2 knockdown on p27 protein levels in 8988T cells expressing a control shRNA or shRNAs to GOT2. **e** and **f**, p27 protein levels in 8988T cells expressing a control shRNA or a shRNA to GOT2 supplemented with or without NAC **e**, OAA or Asp **f**. **g**, Percentages of SA-β-gal positive cells in control and GOT2 knockdown 8988T cells transfected with p27 or control siRNA as indicated. **h**, A proposed model depicting the regulation of PDAC senescence by GOT2. All error bars ± SEM. **p < 0.01 and ***p < 0.001

Next, we investigated the molecular mechanism underlying senescence induction by GOT2 downregulation. The tumor suppressors p53 and p16 have critical roles in cellular senescence^3^. First, to test whether GOT2 knockdown induces senescence through p53-mediated processes, we treated cells with pifithrin-α (PFT-α), a p53 inhibitor. Interestingly, the inhibition of p53 did not influence senescence induction by GOT2 knockdown in 8988T PDAC cells (Fig. 5a and S5A). Consistent with these results, we did not detect the change of messenger RNA (mRNA) levels of p21, a p53 target gene (Fig. 5b). Moreover, when we assessed the protein levels of p21 and p53, we did not observe a clear, coordinated change by GOT2 knockdown (Fig. 5c). Stimuli that drive senescence can also engage the p16-pRb pathway and increased p16 expression contributes to the acquisition of senescence-related phenotypes^3,16^. However, we found that GOT2 knockdown also had no significant effect on p16 mRNA levels (Fig. 5b). Together, these data indicate that GOT2 knockdown may elicit senescence not through classical p53 or p16 pathways in PDAC cells.

The cyclin-dependent kinase inhibitor p27, frequently downregulated in many human cancers, regulates cell cycle progression and also plays a crucial role in the induction and maintenance of senescence^6,7^. When GOT2 was knocked down, p27 mRNA and protein levels were robustly induced in PDAC cells (Figs. 5b, d), implying that p27 might be required for senescence induction by GOT2 knockdown. Because p27 also can be regulated by proteasome-dependent proteolytic pathway^17^, we tested whether GOT2 knockdown has a posttranslational effect on p27 protein stability. However, cycloheximide (CHX) treatment revealed that the increase in p27 abundance following GOT2 knockdown is not due to increased p27 half-life (Fig. S5B).

Given the function of GOT2 in the redox homeostasis, we speculated that GOT2 knockdown increased cellular ROS levels which may elicit p27-mediated senescence. To test this idea, we first investigated whether p27 expression is affected by cellular ROS levels. In the presence of exogenous hydrogen peroxide (H_2_O_2_), p27 is highly induced (Fig. S5C). Next, to confirm that the elevated p27 expression upon GOT2 knockdown was caused by increased ROS levels, we assessed p27 protein levels with NAC. Importantly, NAC treatment significantly reduced p27 expression in GOT2 knockdown cells (Fig. 5e, S5D and E). Consistent with these results, we found that the increase of p27 upon GOT2 knockdown was also rescued by the addition of OAA or Asp (Fig. 5f, S5D and E). Lastly, to directly test whether p27 is required for the GOT2 knockdown-mediated senescence, we suppressed p27 expression using short interfering RNA (siRNA) in 8988T cells and assessed senescence upon GOT2 knockdown. Remarkably, silencing of p27 almost completely diminished senescence induction (Fig. 5g, S5F and G). Collectively, these results support our model that GOT2 knockdown increases ROS levels in PDAC cells, which elicits the p27-mediated cellular senescence (Fig. 5h).

## Discussion

In this study we demonstrate that mitochondrial Gln metabolism via GOT2 regulates senescence in PDAC cells. GOT2 is required to sustain PDAC growth and to repress senescence, probably through maintaining cellular redox balance. We find that GOT2 knockdown results in an elevated ROS production, which leads to p27-dependent senescence. Consistent with previous work showing that PDAC cells are strongly dependent on Gln metabolism^9,10^, our study reveals GOT2 as an important regulator of senescence in PDAC.

Metabolic regulation is intimately involved in determining cell-fate decisions, such as quiescence and proliferation. Emerging evidence has suggested that metabolism plays key roles in senescence pathways. For example, dysregulation of mitochondrial functions causes both growth arrest and senescence-associated secretory phenotype^18^. In progeroid mice, which have mitochondrial DNA mutations and aging phenotypes, senescent cells exhibits rapidly accumulated in inguinal adipose tissues. Recently, NAD^+^ and NADP^+^-linked malic enzymes have been reported to repress replicative senescence^15^. Downregulation of malic enzymes activates p53, leading to induction of senescence in IMR90 cells. As malic enzymes are involved in Gln-dependent NADPH production and ROS regulation, these results support our notion that GOT2-mediated redox balance contributes to senescence regulation.

Our findings are consistent with previous work showing that Gln metabolism is essential for the maintenance of cellular redox state in PDAC^9^. This study identifies the profound impact of GOT2 function on Gln-dependent NADPH production and ROS regulation in PDAC cells (Fig. 4). The enhanced levels of cellular ROS and an altered redox status have been observed in most cancer cells. Increased ROS levels facilitate cancer growth and malignant progression, while at the same time these can cause cellular toxicity. As ROS generation is also intimately linked with both the initiation of PDAC and Ras-induced tumor growth^2,19^, a delicate regulation of intracellular ROS levels is essential for PDAC development and progression. Thus, this may explain why GOT2 expression is increased in human pancreatic cancers^13^ and PDAC cells are markedly sensitive to GOT2 inhibition, whereas it is dispensable in non-transformed cells.

Previous studies suggest that pancreatic cancer develops through the successive accumulation of mutations^2^. In addition to KRAS, the key regulators of classical senescence pathways, such as p53 and p16, are the most frequently mutated proteins in human PDAC^20^. Likewise, genetically engineered mouse models of PDAC, which recapitulate the human disease, have Kras activation and combinations of inactivating mutation in p53 and p16 to develop pancreatic cancer^2^. Therefore, it is likely that mutations of these genes may permit PDAC cells to bypass the classical senescence pathways and contribute to the therapeutic resistance of PDAC. In support of this idea, GOT2 knockdown did not induce significant differences in p53, p21, and p16 expression (Figs. 5b, c). Importantly, we find that knockdown of GOT2 induces p27-dependent premature senescence in PDAC (Fig. 5g), highlighting the potential importance of this GOT2-mediated senescence pathway for developing therapeutic approaches for PDAC.

Our studies demonstrate that elevated ROS in the inhibition of GOT2 induces p27 expression. pRb has been shown to play critical roles in cell cycle arrest in response to ROS and increases p27 protein levels by inhibiting Skp2-medaited protein degradation^21,22^. However, we observe that the induction of p27 following GOT2 knockdown primarily occurs through a transcriptional mechanism but not through proteasome-dependent degradation in PDAC cells (Fig. 5b and S5B). Several transcription factors are known to regulate p27 expression. For example, transcription of *p27* gene is activated in part by forkhead box class O family (FoxO) proteins^21,23^, a conserved family of transcription factors involved in metabolism, cell cycle progression and cellular redox homeostasis^24,25^. Interestingly, several groups have provided evidence that FoxO transcriptional activity is enhanced by cellular oxidative stresses^21,25–27^. Thus, it will be interesting for future studies to examine whether FoxO transcription factors regulate growth and senescence of PDAC cells, in part via modulating p27 expression and/or cellular ROS levels.

In this study we demonstrate that mitochondrial GOT2 serves to control senescence in PDAC cells through regulation of ROS, suggesting that targeting the unique redox regulatory pathways of PDAC might be an effective strategy to treat these cancers.

## Material and methods

### Cell culture

HEK293T, 8988T and other human PDAC cell lines were cultured in Dulbecco’s modified Eagle’s medium (Biowest, Nuaillé, France) supplemented with 10% fetal bovine serum (FBS, Biowest) and penicillin/streptomycin (Biowest). WI38 cells were cultured in MEM media (Hyclone, Logan, UT, USA) with 10% FBS and antibiotics. HPDE cells were cultured as described previously^9^.

### Constructs and reagents

The following antibodies were used: GLS (Abcam, Cambridge, MA, USA), GOT2 (Cusabio biotech, Wuhan, China), p27 (BD biosciences, Franklin Lakes, NJ, USA), p53 (Santa cruz Biotechnology, Dallas, USA), p21 (Abcam) and β-actin (Sigma, St. Louis, MO, USA). NAC, DON, EGCG, AOA, PFT-α and hydrogen peroxide were purchased from Sigma. Information about all shRNA vectors were described previously^9^. The human GOT2 was cloned into retroviral pBabe vector and used to generate stable cell line.

### Cell viability assay

Cells were plated into 96-well plates at 1000 cells per well in 100 μl of growth media. The following day, growth media was replaced with that containing oxaloacetate (4 mM) or Aspartate (4 mM). Parallel plates were analyzed at 3 days by Cell Titer Glo analysis (Promega, Fitchburg, WI, USA), per the manufacturer’s instruction.

### Western blotting

Cells were lysed with lysis buffer (150 mM NaCl, 50 mM Tris-HCl, pH 7.5 and 0.5 % NP-40) supplemented with protease inhibitor cocktail (Roche, Diagnostics, Mannheim, Germany) and phosphatase inhibitors (Sigma). Cell lysates were separated by sodium dodecyl sulfate-polyacrylamide gel electrophoresis and immunoblotting.

### Quantitative RT-PCR

Total RNA was prepared with Ribospin kit (GeneAll, Seoul, Korea) according to the manufacturer’s instructions. 0.5 μg of total RNA was reverse-transcribed using iScript cDNA synthesis kit (Bio-Rad, Hercules, CA, USA). Diluted cDNAs were analyzed by real-time PCR using SYBR Green master mix on a Light Cycler 480 (Roche). The level of gene expression was normalized to β-actin. The primer sequences were: GCCCTCCCCAGTCTCTCTTA and TCAAAACTCCCAAGCACCTC for p27; GGCAGACCAGCATGACAGATTT and GGCGGATTAGGGCTTCCTCT for p21; GTGGACCTGGCTGAGGAG and CTTTCAATCGGGGATGTCTG for p16; TTCCAGAAGGCACAGACATG and GGCTCAGTACTCTTTCACCAG for GLS1; AGGAATGACACCAGGGTTTG and TCAGACTCACCAACAGCAATAC for GLUD1; GTTTGCCTCTGCCAATCATATG and GAGGGTTGGAATACATGGGAC for GOT2; CATGGACATTGTCTGAACC and TTACCCAGGACCGACTCCTT for GPT2; and CTACGTCGCCCTGGACTTCGAGC and GATGGAGCCGCCGATCCACACGG for human actin.

### β-galactosidase (β-gal) staining

β-gal staining was performed as previously described^28^. Cells were washed two times with PBS and fixed with 4% formaldehyde for 5 min. And then cells were incubated at 37 °C with β-gal staining solution for overnight. Stained cells were evaluated using an inverted phase contrast microscope (Olympus, Center Valley, PA, USA). Percentages of stained cells were calculated randomly at least 300 cells.

### Immunofluorescence

Cells were grown on coverslips in a 6-well plate and were fixed in methanol for 5 min at room temperature (RT). After PBS washing, cells were permeablized for 20 min on 0.5% PBST (PBS containing 0.5% Triton X-100). The permeabilized cells were washed with PBS twice and blocked in 0.1% PBST (PBS containing 0.1% Triton X-100) with 5% normal goat serum (NGS) for 30 min. Then the cells were incubated with PML antibody (Santa Cruz) in 0.1% PBST with 5% NGS overnight at 4 °C. After washing in 0.1% PBST, cells were stained with FITC-conjugated secondary antibody for 1 h at RT. Finally, cell were washed five times in 0.1% PBST and mounted with Vectashield mounting medium with DAPI (Vector Laboratories, Burlingame, CA, USA). The fluorescence signal was detected using confocal microscopy.

### ROS determination

The levels of cellular ROS were assessed by using DCFDA (Invitrogen, Grand Island, NY, USA) according to the manufacturer’s instructions. Cells were washed with PBS and then incubated at 37 °C for 20 min in Hank’s balanced salt solution (Gibco, Big Cabin, OK, USA) containing 5 μM DCFDA. Cells were trypsinized and resuspended in Hank’s balanced salt solution. Fluorescence was measured by flow cytometry using a FACSCanto (BD biosciences).

### NADPH/NADP^+^ ratio

NADPH/NADP^+^ ratios were measured using the NADP/NADPH-GLO assay kit (Promega) following the manufacturer’s instructions. Briefly, cells were lysed in base solution 1% dodecyl trimethyl azanium bromide (Sigma). Cell lysates were mixed with NADP/NADPH-Glo detection reagents. After 30 min, NADPH/NADP^+^ ratios were measured using luminometer.

### Statistical analysis

Unpaired two-tailed Student’s *t* test was performed unless otherwise noted. All experiments were performed at least two or three times.

## Electronic supplementary material

supplemental text

supplemental figure
