# Supplementary material for: Mitochondrial glutamine metabolism via GOT2 supports pancreatic cancer growth through senescence inhibition
Source: Cell Death Dis. 2018 Jan 19;9(2):55. doi: 10.1038/s41419-017-0089-1 (PMC5833441; doi:10.1038/s41419-017-0089-1)
Supplement: Supplementary file 1 — supplemental text [file 41419_2017_89_MOESM1_ESM.docx]

**Supplementary figure legend**

**Figure S1**. Inhibition of mitochondrial glutamine metabolism induces cellular senescence. (A and B) Representative images of SA β-gal staining for 8988T cells treated with DON (A) and expressed a shRNA to GLS (B).

**Figure S2.** Mitochondiral transaminase GOT2 regulates PDAC senescence. (A) Representative images of SA-β-gal positive cells treated with EGCG or AOA. (B) Representative images of SA β-gal staining (left) and relative mRNA levels of GLUD1 (right) of 8988T cells expressing a control shRNA (shGFP) or two independent shRNAs targeting GLUD1. (C) Representative images of SA β-gal staining (left) and relative mRNA levels of GOT2 (right) of 8988T cells expressing a control shRNA or shRNAs to GOT2. (D) GOT2 protein levels in whole-cell lysates from vector and GOT2 overexpressed 8988T cells expressing a control shRNA or shRNAs to GOT2 (left). β-actin serves as a loading control. Representative images of SA β-gal staining of vector or GOT2-overexpressed 8988T cells expressing a control shRNA or shRNAs to GOT2 (right). (E) Survival of 8988T cells expressing a control shRNA or shRNAs to GOT2. Cell viability was measured via PI exclusion assay. (F) Relative mRNA levels in 8988T cells expressing a control shRNA or a shRNA to GPT2. (G) Percentages of SA-β-gal positive cells in control and GPT2 knockdown 8988T cells. All error bars ± SEM.

**Figure S3.** GOT2 functions as an important regulator of senescence in PDAC but not in non-transformed cells. (A) Representative images of SA β-gal staining for Panc1, PL45 and Tu8902 cells expressing a control shRNA or two independent shRNAs targeting GOT2. (B) Representative images of SA β-gal staining of HPDE, WI38 and HEK293T cells expressing a control shRNA or shRNAs to GOT2.

**Figure S4.** GOT2 functions as an important regulator of senescence in PDAC but not in non-transformed cells. (A) Relative levels of Asp and OAA in 8988T cells expressing a control shRNA or two independent shRNAs targeting GOT2. Metabolites levels were normalized to protein content. (B) Representative images of SA β-gal staining for 8988T cells expressing a control shRNA or shRNAs to GOT2 with or without NAC. (C) Representative images of SA β-gal staining for control or AOA treated 8988T cells cultured with or without NAC. (D) Representative images of SA β-gal staining for 8988T cells expressing a control shRNA or shRNAs to GOT2 with or without OAA. (E and F) Senescence induction (E) and representative images of SA β-gal staining (F) of 8988T cells expressing a control shRNA or shRNAs to GOT2 supplemented with or without Asp. All error bars ± SEM. *p< 0.05 and **p< 0.01.

**Figure S5.** p27 mediates GOT2 knockdown induced senescence. (A) Representative images of SA β-gal staining for control or PFT-α treated 8988T cells. (B) p27 protein levels in whole-cell lysates from 8988T cells expressing a control shRNA (shGFP) or a shRNA to GOT2 treated with or without cycloheximide. β-actin serves as a loading control. (C) Western blot for p27 in 8988T cells treated with or without hydrogen peroxide (H_2_O_2_). (D and E) p27 protein levels in Panc1 (D) and PL45 (E) cells expressing a control shRNA (shGFP) or a shRNA to GOT2 supplemented with or without OAA or NAC. (F) Western blot for p27 in 8988T cells expressing a control shRNA or shRNAs to GOT2 transfected with sip27 or control siRNA. (G) Representative images of SA β-gal staining in control and GOT2 knockdown 8988T cells transfected with sip27 or control siRNA as indicated.

**Supplementary material and methods**

**Metabolites measurement**

Cells were grown to about 60% confluence on 10-cm dishes in biological triplicate. After 24h, cells were harvested in ice-cold methanol/H_2_O (80/20, v/v). Metabolites were extracted from the aqueous phase by liquid-liquid extraction after adding chloroform. The samples were reconstituted with 50% methanol prior to LC-MS/MS methods. The LC-MS/MS system was equipped with an Agilent 1290 HPLC (Agilent), Qtrap 5500 (ABSciex), and reverse phase column (Synergi fusion RP 50 × 2 mm). Multiple reaction monitoring was used in the negative ion mode and the extracted ion chromatogram (EIC) corresponding to the specific transition for each metabolite was used for quantitation. Area under the curve of each EIC was normalized to that of EIC of internal standard. Metabolites levels were normalized to protein content, which was determined by performing a BCA protein assay (Thermo scientific) of cells cultured identically to the experimental cells.

**Flow cytometric measurement**

Cells at less than 80% confluence were treated with DNA damage agents. After treatment, cells were harvested by trypsinization, pelleted by centrifugation, and resuspended in PBS containing 3% fetal bovine serum. The measurement of cell death was performed by flow cytometry using propidiumiodide (PI) staining, as previously described[^18^](#_ENREF_18).
