# Supplementary figures and images for: Mitochondrial glutamine metabolism via GOT2 supports pancreatic cancer growth through senescence inhibition
Source: Cell Death Dis. 2018 Jan 19;9(2):55. doi: 10.1038/s41419-017-0089-1 (PMC5833441; doi:10.1038/s41419-017-0089-1)

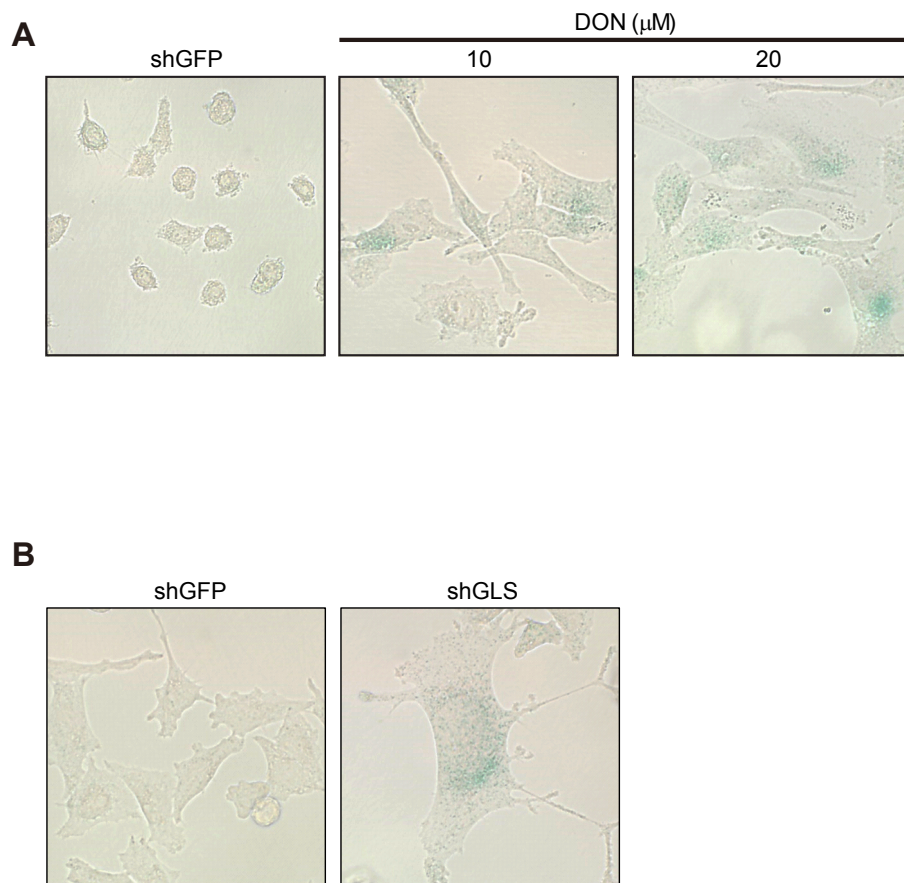

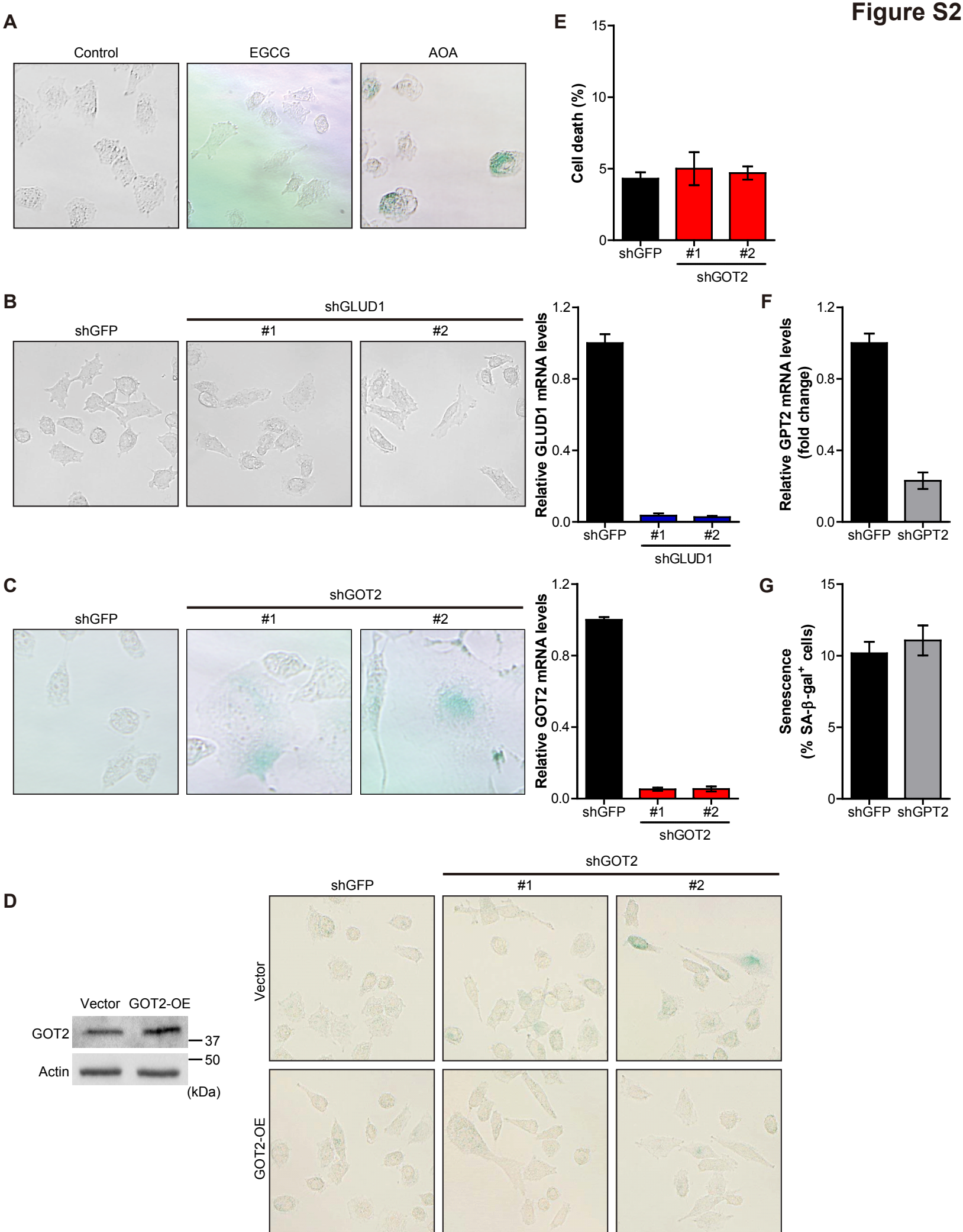

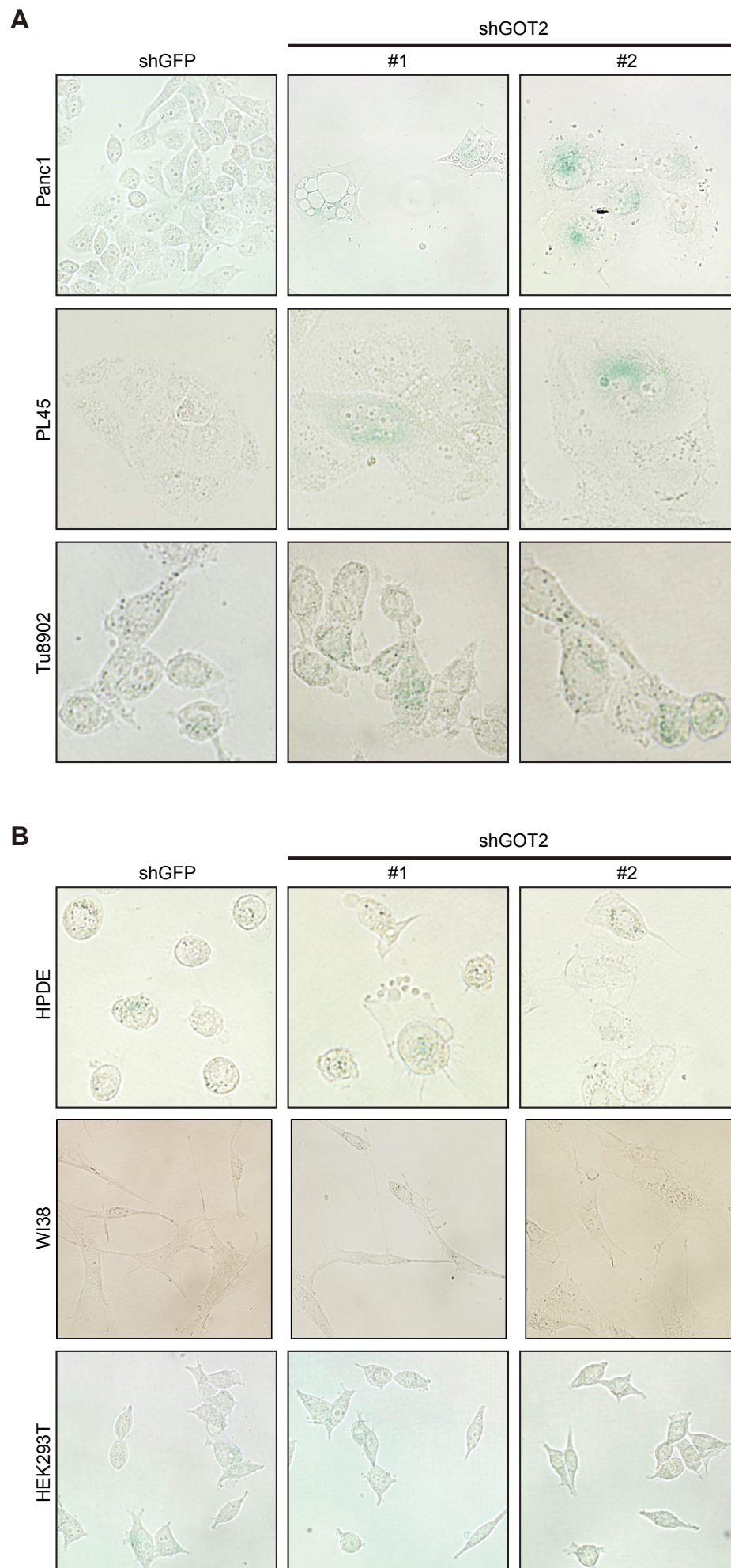

**A**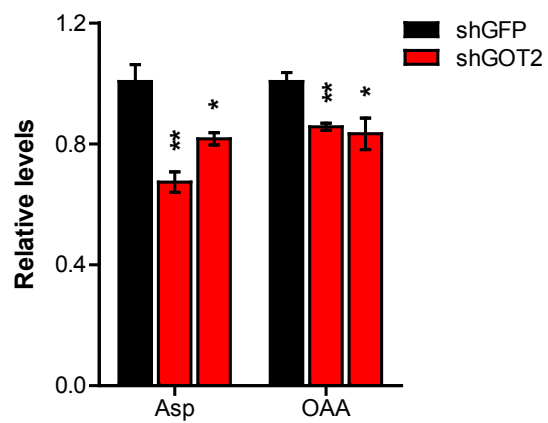**B**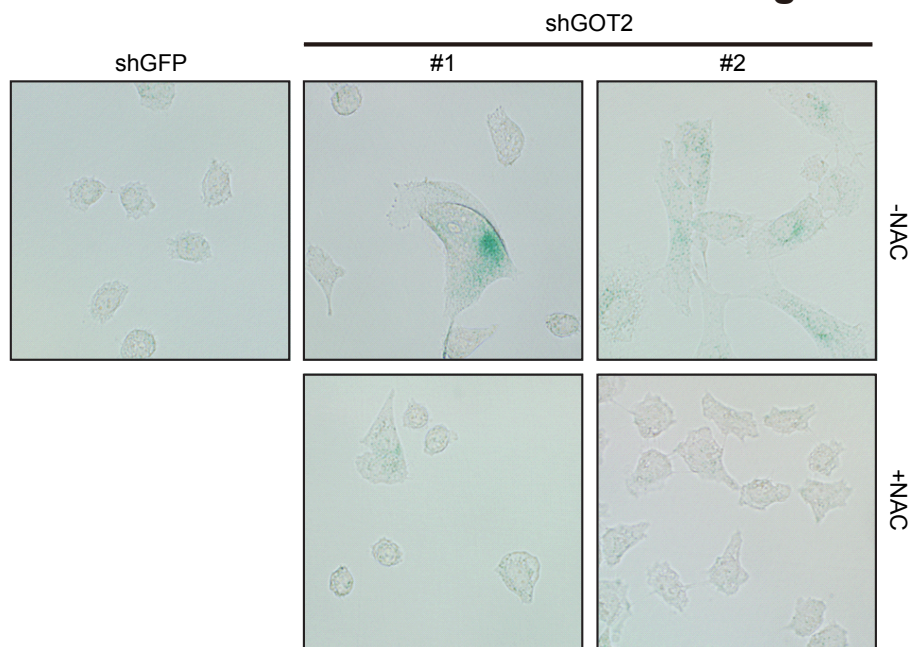**C**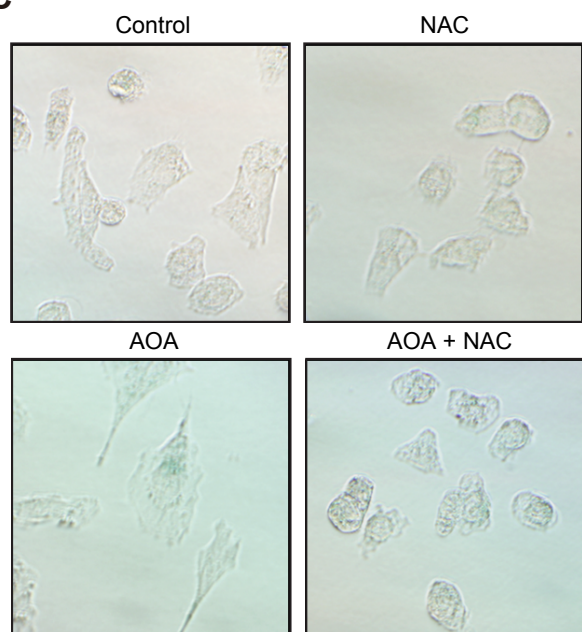**D**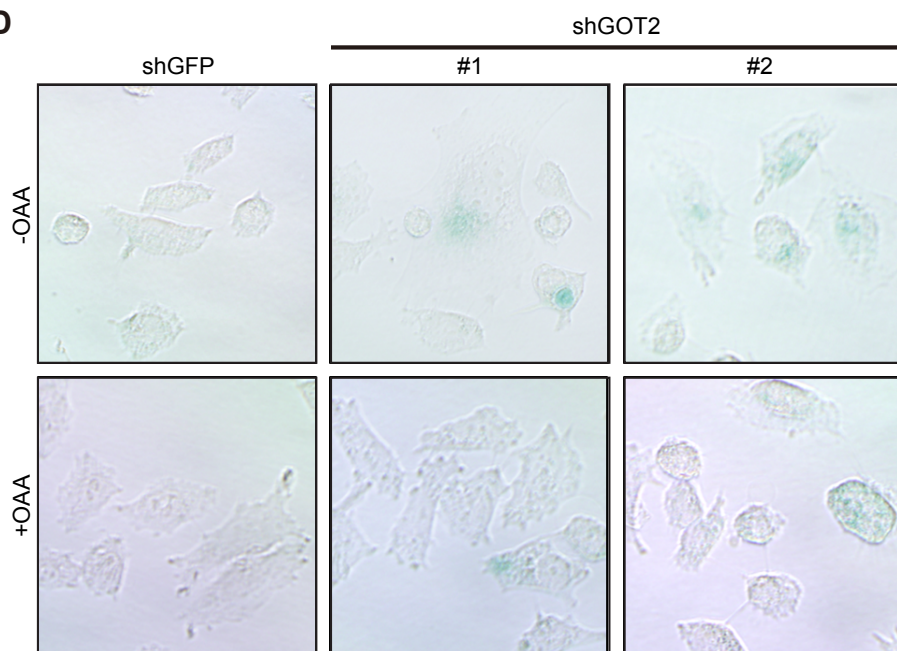**E**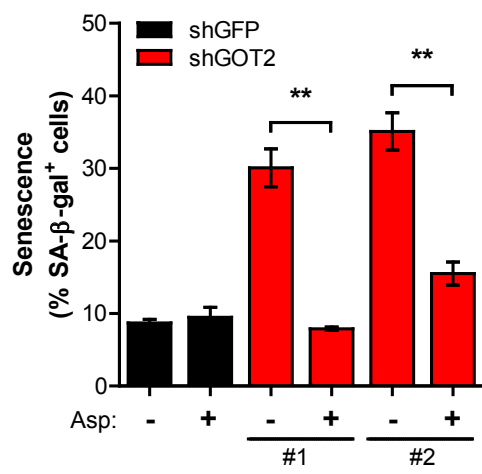**F**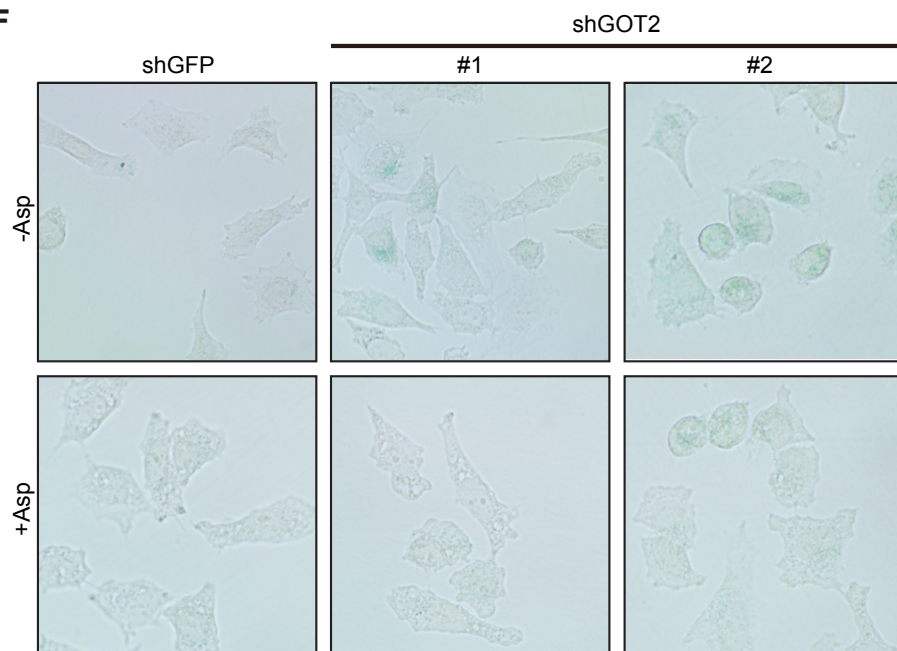

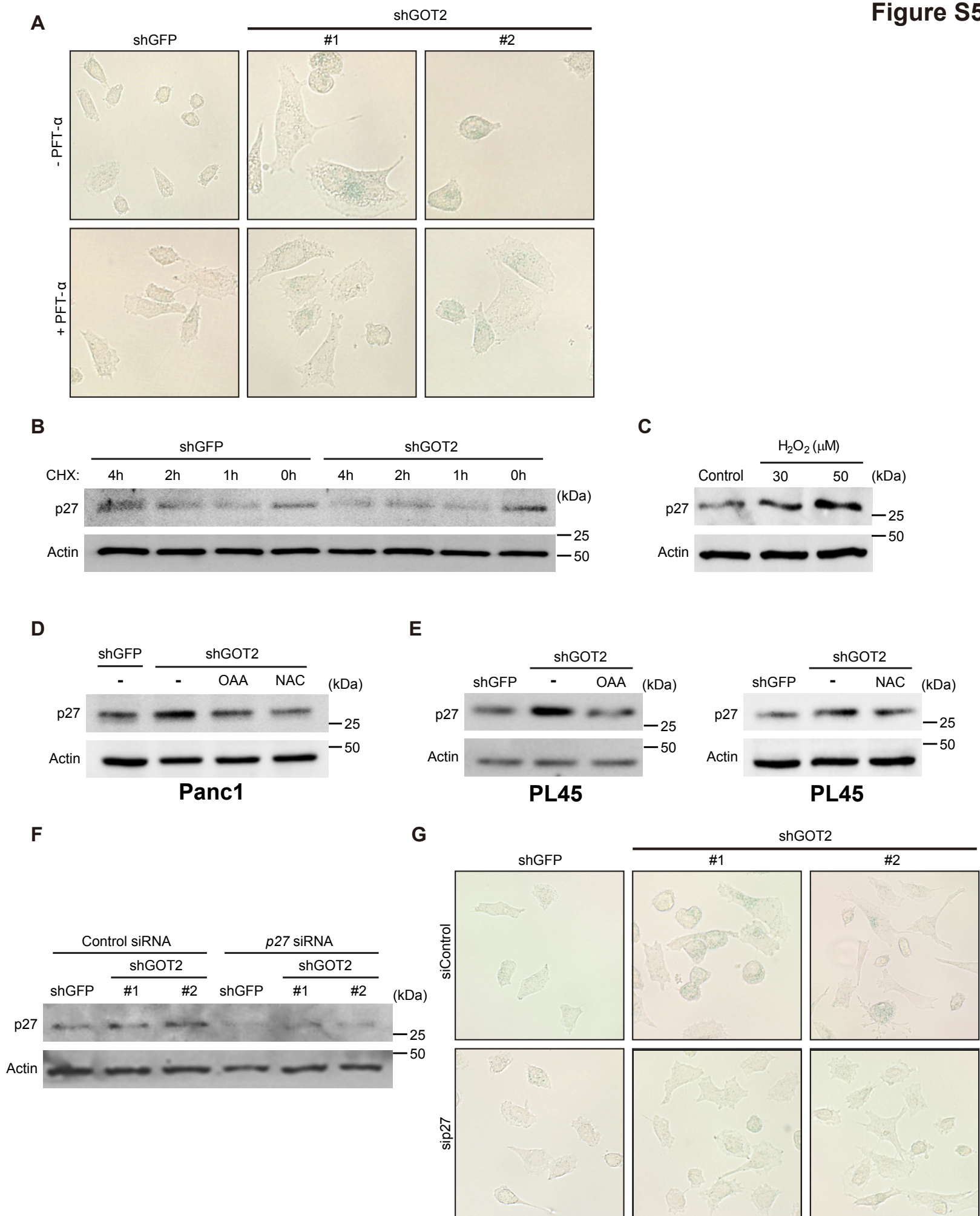

Supplement: Supplementary file 2 — supplemental figure [file 41419_2017_89_MOESM2_ESM.pdf]
